# Cytokine Profiles and Inflammatory Implications in Chagas Disease: Associations with Ventricular Function and Conduction Disorders

**DOI:** 10.3390/medsci13040309

**Published:** 2025-12-08

**Authors:** Mario Principato, Maria Victoria Carvelli, Analia Gladys Paolucci, Silvia Miranda, Guillermo Alberto Keller, Manuel Lago, Guillermo Di Girolamo, Justo Carbajales

**Affiliations:** 1 Cardiology Division, Hospital General de Agudos José María Ramos Mejía, Buenos Aires C1221ADC, Argentina; 2 Instituto Alberto C Taquini de Investigación en Medicina Traslacional (IATIMET), Facultad de Medicina, Universidad de Buenos Aires, Buenos Aires C1122AAJ, Argentina; simir2@gmail.com (S.M.); gkeller@fmed.uba.ar (G.A.K.)

**Keywords:** cytokines, Chagas disease, ventricular dysfunction, intraventricular conduction disorders

## Abstract

Background: The roles of cytokines and chemokines in the pathogenesis of Chagas cardiomyopathy (CC) have been proposed, yet their clinical significance with respect to conduction disturbances and left ventricular ejection fraction (LVEF) remains unclear. Aim: The objective of this study was to analyze the associations between cytokine levels and systolic function, comparing patients with preserved and reduced ejection fractions. As a secondary objective, we evaluated whether differences were present in cytokine levels within the subgroup with preserved ejection fraction, depending on the presence or absence of intraventricular conduction disturbances. Methods: We conducted an analytical cross-sectional study involving patients with Chagas disease and a healthy control group. Among patients with Chagas disease, those with preserved (>50%) and reduced (<35%) left ventricular ejection fraction (LVEF) were selected. The preserved-LVEF group included individuals with and without conduction disorders. Cytokines (IFN-γ, IL-1β, IL-6, IL-10, IL-12p70, IL-15, IL-17A, MCP-1, MIP1α, TNF-α, and IL-2) were quantified using a magnetic bead-based multiplex assay. Results: Forty-four patients with CD (26 men, 59%) and 14 seronegative controls were included. In the CD group, 50% (n = 22) had preserved LVEF (LVEF > 50%), and 50% (n = 22) had decreased LVEF (≤35%). No significant differences in cytokine concentrations were observed between patients with preserved and reduced LVEF for TNF-α (19.74 ± 8.32 vs. 22.23 ± 6.40 pg/mL; *p* = 0.189), IL-6 (2.17 ± 2.41 vs. 5.40 ± 6.40 pg/mL; *p* = 0.145), IL-2 (2.61 ± 1.05 vs. 2.97 ± 1.79 pg/mL; *p* = 0.481), MCP-1 (214.18 ± 96.99 vs. 183.83 ± 63.21 pg/mL; *p* = 0.481) and IFN-γ (9.04 ± 4.90 vs. 7.64 ± 3.78 pg/mL; *p* = 0.372). Within the subgroup with preserved LVEF (n = 22), those with conduction disorders (n = 10) exhibited higher levels of IL-10 (24.49 vs. 9.83 pg/mL; q = 0.009), IL-12p70 (13.20 vs. 9.02 pg/mL; q = 0.027), IL-2 (2.70 vs. 2.07 pg/mL; q = 0.023), IL-15 (7.09 vs. 3.36 pg/mL; q = 0.018), MIP1α (10.33 vs. 3.35 pg/mL; q = 0.014) and IFN-γ (10.83 vs. 7.25 pg/mL; q = 0.005), compared to those without conduction disorders (n = 12). Notably, patients with CD and preserved LVEF (>50%) without conduction disturbances presented cytokine profiles similar to those of seronegative healthy controls with LVEF ≥ 50%. Conclusions: Elevated levels of specific cytokines were associated with conduction disturbances in patients with preserved LVEF. Despite this finding, a causal relationship cannot be established, and future studies are needed to explore their prognostic or therapeutic significance.

## 1. Introduction

Chagas disease (CD) is a neglected tropical disease caused by the obligate intracellular protozoan parasite Trypanosoma cruzi [1]. Initially endemic to Central and South America, the disease now affects an estimated 10 million people across all continents, primarily due to migration [1,2]. The disease progresses through three distinct phases. Following the initial acute symptomatic phase, patients enter an indeterminate asymptomatic phase characterized by a positive serology for CD and a low parasite load. Approximately one-third of patients progress to the chronic phase, typically 10–20 years after the primary infection. The most common clinical manifestation of the chronic phase is Chagas cardiomyopathy (CC) [2,3]. The histopathology of the heart in CC is characterized by diffuse myocytolysis, fibrosis, and cellular necrosis [1,4,5,6], leading to left ventricular systolic dysfunction, conduction disturbances, heart failure, and sudden cardiac death [7]. One intriguing hypothesis is that interleukin-mediated processes play a role in the disease pathogenesis. Strong and significant inverse associations have been reported between levels of inflammatory cytokines (IFN-γ, TNF-α, and IL-6) and left ventricular function. Conversely, IFN-γ and TNF-α have demonstrated weaker direct associations with diastolic diameter. However, far less is known about the role of IL-10 and other regulatory cytokines in cardiac function.

The mechanisms underlying the development of CC in only 15–30% of patients with CD remain poorly understood. Recent hypotheses include a potential role for autoantibodies and molecular mimicry [8,9]. Another hypothesis proposes that cytokines play a critical role in disease progression. An association has been reported between circulating inflammatory cytokines (IFN-γ, TNF-α, and IL-6) and ventricular dysfunction due to other etiologies [10,11,12]. However, the roles of other cytokines—such as IL-10, IL-12, IL-15, IL-17, and MCP-1—in CC have not been thoroughly investigated.

Aim: The objective of this study was to analyze the associations between cytokine levels and systolic function, comparing patients with preserved and reduced ejection fractions. As a secondary objective, we analyzed whether differences existed in cytokine levels within the subgroup with preserved ejection fraction, depending on the presence or absence of intraventricular conduction disturbances.

## 2. Materials and Methods

Design and Population: An analytical cross-sectional study was conducted in patients with CD with altered left ventricular ejection fraction (LVEF), CD patients with preserved LVEF, and healthy volunteers.

Sample: Inclusion criteria were patients aged 21–80 years, seropositive for CD for more than 20 years (certified by treating physician or confirmed by serology), presumed to have been infected in an endemic area, and presenting an LVEF ≤ 35% or ≥50% within the previous 12 months. Patients with a pacemaker were included if the pacing rate was <50%. Exclusion criteria were patients who were unwilling to sign informed consent; those with severe comorbidities (other than cardiovascular disease) and a life expectancy <1 year; those who had participated in other research studies within the previous 30 days; those with suspected poor follow-up reliability, alcohol or drug abuse within the previous 6 months; clinical or laboratory evidence of liver disease (transaminases > 3 × upper limit of normal, bilirubin > 2); LVEF between 36% and 49% (excluded to avoid overlap; those receiving medications known to affect cardiovascular outcomes (immunosuppressants, nitrates, estrogens, statins, NSAIDs, corticosteroids), coronary artery disease (acute or chronic) with revascularization, coronary angioplasty, or bypass within the prior 6 months; patients with advanced renal disease (creatinine > 3 mg/dL), severe chronic obstructive pulmonary disease (COPD), other forms of dilated cardiomyopathy (e.g., associated with rheumatoid arthritis, diabetes, hypertension), or significant valvular disease (except that due to annular dilation); and patients with a pacemaker rate > 50% or with autoimmune diseases (e.g., lupus, scleroderma, hepatitis C). As a healthy control group, a number of health professionals were chosen, who work in the same healthcare center, with ages similar to the group of Chagas patients, without associated cardiac pathology.

Procedures: After providing informed consent, patients underwent a complete medical history, physical examination, and relevant diagnostic and therapeutic evaluations. LVEF was confirmed by echocardiography. A 12-lead ECG was performed for assessment purposes. The electrocardiograms were evaluated in a blinded manner by two independent cardiologists. Intraventricular conduction delays (IVCDs) were defined as right bundle branch block (RBBB), left anterior fascicular block (LAFB), or left bundle branch block (LBBB).

Blood Sampling and Cytokine Analysis: Six-milliliter aliquots of fasting venous blood were collected in EDTA Vacutainer tubes (Becton Dickinson, Franklin Lakes, NJ, USA). Samples were centrifuged immediately at 1200× *g* for 10 min, and a freshly prepared protease inhibitor cocktail (P8340, Sigma-Aldrich, Burlington, MA, USA) was added (1% *v*/*v*). Plasma aliquots were stored at –20 °C until analysis. The concentrations of IFN-γ, IL-1β, IL-6, IL-10, IL-12p70, IL-15, IL-17A, MCP-1, MIP1α, TNF-α, and IL-2 were measured using a magnetic bead-based multiplex assay (HCYTOMAG-60K, Merck Millipore, MO, USA) run on a Magpix^®^ 4.3 platform. All samples were analyzed in duplicate. Standards were included in each run, and concentrations were calculated using xPONENT^®^ software v4.2 (results expressed in pg/mL). All patients received standard cardiovascular therapy according to contemporary clinical guidelines.

Statistical Analysis: Nonparametric statistical methods were applied. Categorical variables were analyzed using the chi-square test and Cramér’s V, and odds ratios were calculated when applicable. The Mann–Whitney U test and the Kolmogorov–Smirnov Z test were used for comparisons between categorical and metric variables. The significance threshold was set at *p* < 0.05.

The magnitude of the difference between the groups is expressed as the Hodges–Lehmann position statistic and its corresponding 95% confidence interval. Only null hypotheses with a *p*-value ≤ the q-value calculated using the Benjamini–Hochberg procedure for the false discovery rate were rejected; therefore, differences between groups for cytokines 1 to 6 in the ranking were considered significant. R software version 4.3.1 was used.

Ethics and Confidentiality: Patients were identified by their initials followed by a code corresponding to their group assignment. The medical staff responsible for patient care retained the link between identifiers and patient names, in accordance with medical confidentiality requirements and the Argentine Personal Data Protection Law No. 25326. Patients received a copy of their clinical evaluations and, upon request, their genetic data. They were free to withdraw from the study at any time without affecting their medical care.

Clinical trial registration number: IS005097 (National Registry of Health Research, available at https://sisa.msal.gov.ar/sisa/ last accessed on 10 October 2025).

## 3. Results

**Figure 1:**
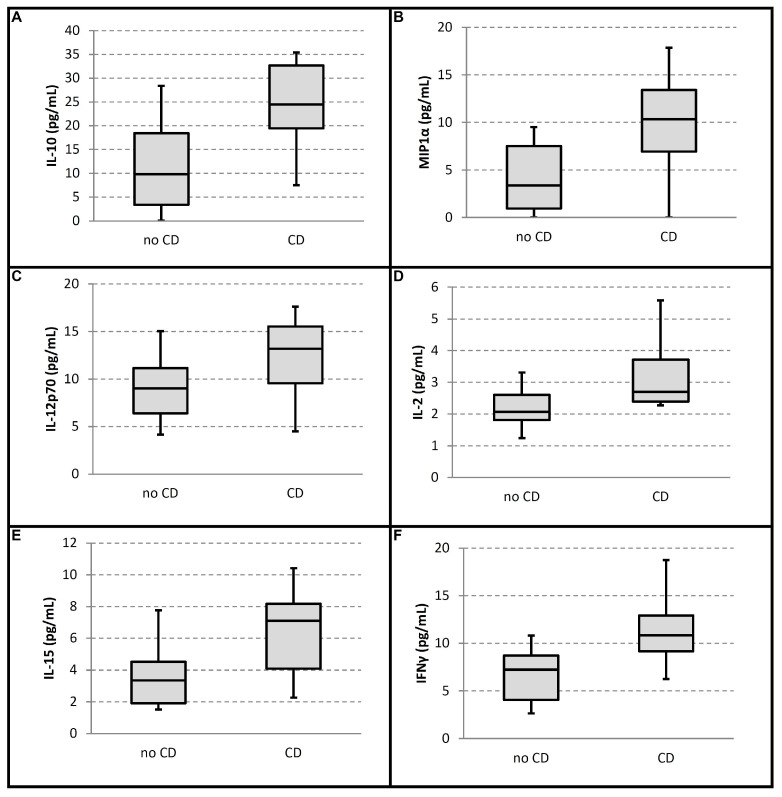
Comparison of cytokine levels in patients with preserved LVEF with and without intraventricular conduction disturbances: (**A**) IL-10; (**B**) MIP1α; (**C**) IL-12p70; (**D**) IL-2; (**E**) IL-15; (**F**) IFNγ. In all panels, the box indicates the median (25th to 75th percentile). The vertical lines indicate the min–max range. For better visual identification, dark gray boxes indicate patients with conduction disorders, while light gray boxes indicate patients without conduction disorders.

The sample included 44 patients with CD (26 men, 59%) and 14 seronegative controls (healthy volunteers). In the CD group, 50% (n = 22) had preserved LVEF (>50%), and 50% (n = 22) had reduced LVEF (≤35%). All selected patients (normal and reduced LVEF) were in functional class 1; therefore, their clinical presentation is not described. The description of LVEF was preferred over ventricular diameters because the latter has greater prognostic value. The mean LVEF was 60.9% (SD 6.68) in the group with preserved function and 28.8% (SD 4.38) in those with systolic dysfunction. All patients with reduced LVEF exhibited some degree of intraventricular conduction disturbance: right bundle branch block (RBBB), left anterior fascicular block (LAFB), or complete left bundle branch block (LBBB). The baseline characteristics of the study population are presented in Table 1. Serum cytokine levels (Table S1) in patients with preserved versus reduced LVEF are shown in Table 2 and Figure 1. Patients with reduced LVEF exhibited slightly higher levels of TNF-α (22.23 ± 6.40 vs. 19.74 ± 8.32 pg/mL; *p* = 0.189), IL-6 (5.40 ± 6.40 vs. 2.17 ± 2.41 pg/mL; *p* = 0.145), and IL-2 (2.97 ± 1.79 vs. 2.61 ± 1.05 pg/mL; *p* = 0.481). In contrast, patients with preserved LVEF had slightly higher levels of MCP-1 (214.18 ± 96.99 vs. 183.83 ± 63.21 pg/mL; *p* = 0.481) and IFN-γ (9.04 ± 4.90 vs. 7.64 ± 3.78 pg/mL; *p* = 0.372).

Within the subgroup with preserved LVEF (n = 22), as shown in Table 3, individuals with conduction disorders (n = 10) had higher levels of IL-10 (24.49 vs. 9.83 pg/mL; q = 0.009), IL-12p70 (13.20 vs. 9.02 pg/mL; q = 0.027), IL-2 (2.70 vs. 2.07 pg/mL; q = 0.023), IL-15 (7.09 vs. 3.36 pg/mL; q = 0.018), MIP1α (10.33 vs. 3.35 pg/mL; q = 0.014) and IFN-γ (10.83 vs. 7.25 pg/mL; q = 0.005), compared to those without conduction disorders (n = 12).

Notably, patients with CD and preserved LVEF (>50%) who did not exhibit conduction disturbances showed cytokine profiles similar to those of seronegative healthy controls with LVEF ≥ 50%.

## 4. Discussion

Chagas disease, caused by *Trypanosoma cruzi*, can progress to a chronic cardiomyopathy characterized by structural disorders, persistent inflammation, and electrophysiological abnormalities. One of the most common findings at this stage is the presence of intraventricular conduction disorders, which can herald systolic dysfunction and progression to heart failure. It has been proposed that the immunopathogenesis underlying these alterations involves a specific activation of the immune system, with increased production of certain proinflammatory and regulatory cytokines. In our study, we observed that among patients with preserved LVEF, those with ventricular conduction disorders had significantly higher levels of specific cytokines compared with those without these disorders. It is therefore worth highlighting certain differences between conduction tissue and contractile myocardium that may explain these findings.

Cardiac conduction tissue is histologically and functionally adapted to generate and transmit electrical impulses, while the contractile myocardium is designed to generate force. This specialization renders the conduction system more vulnerable to metabolic imbalances, ischemic injuries, and pharmacological effects, which may explain why many arrhythmias originate from alterations in these cells rather than in contractile myocardium.

In chronic Chagas disease, one of the most characteristic findings is the early involvement of the cardiac conduction system, even before significant compromise of the contractile myocardium is evident. This selectivity has anatomical, functional, immunological, and pathophysiological bases. The factors determining the susceptibility of conduction tissue to damage include high functional specialization, a lower functional reserve capacity, a greater dependence on specific ion channels, and reduced vascularization compared with contractile myocardium. Cells of the conduction system (such as those of the AV node, His bundle, and Purkinje fibers) are highly dependent on specific ion channels and have a lower functional reserve, especially when exposed to electrical remodeling and inflammatory alterations. The disease induces a significant reduction in the expression of ion-channel subunits, namely Nav1.5 (Na^+^ channel), Kv4.3, Kv3.4, Kir2.1 (K^+^ channels), and L-type Ca^2+^ channels, and significantly reduced expression of Cx40 and Cx43 (gap-junction connexins), which affects electrical conduction in the heart, including Purkinje fibers [13,14,15,16].

*T. cruzi* infection induces a complex and multifaceted immune response in the host, which is essential to understanding how host immune remodeling can trigger cellular remodeling in cardiomyocytes and contribute to global electrical and mechanical remodeling in chronic Chagas cardiomyopathy (CCM).

Our group has been studying the role of specific cytokines in patients with conduction abnormalities [17], in which a Th1-mediated response has been previously implicated in the development of Chagas cardiomyopathy [18,19].

The present study suggests that specific cytokine levels are associated with LVEF and conduction disturbances in patients with CC. Since the publication of the BENEFIT trial, which found no significant differences in clinical outcomes following benznidazole treatment [20], attention has shifted from parasite-associated pathogenicity to host-driven immunopathology, with a focus on potential prognostic markers and therapeutic targets for CC.

No significant differences were observed between patients with preserved and those with reduced EF. Similar findings have been reported in other forms of cardiomyopathy [10,11,12,21,22,23], suggesting that these markers may be less specific to CC.

In vitro studies have demonstrated that TNF-α increases oxidative stress and disrupts mitochondrial membrane integrity [7,24,25], which may help explain its tendency toward higher levels in patients with reduced LVEF.

Conversely, IFN-γ tended to be higher in patients with preserved LVEF. Findings from previous studies have been mixed: some have associated IFN-γ with severe ventricular dysfunction [17,26,27], whereas others have observed decreased IFN-γ-producing lymphocytes in patients without overt cardiac disease [20,28]. Animal studies have shown that IFN-γ can downregulate the expression of contractile proteins [29,30], suggesting its role may vary across disease stages.

In our study, patients with preserved EF and IVCD exhibited elevated levels of IL-10, IL-12p70, IL-2, IL-15, MIP1α, and IFN-γ compared with those with preserved EF and no conduction disturbances. These cytokines have been implicated in the progression from the indeterminate to the chronic phase of CD and may serve as biomarkers for disease progression [7,14,19,31,32,33,34]. Notably, MIP1α has been shown to enhance macrophage trypanocidal activity via increased nitric oxide (NO) production [24]. Further studies are needed to clarify the role of these cytokines in EF reduction, conduction disturbances, or both. Our work is the first to demonstrate differences between patients with and without conduction disorders during the phase in which ejection fraction remains preserved.

IFN-γ is a central cytokine in the Th1-type immune response against *T. cruzi*, but its chronic activity leads to persistent immune-cell infiltration and oxidative stress due to the massive production of NO and reactive oxygen species, such as peroxynitrite (ONOO^−^). NO and ONOO^−^ induce mitochondrial dysfunction, reducing ATP production and altering myocyte energy metabolism. NO also promotes myocardial fibrosis by activating fibroblasts and stimulating extracellular matrix deposition, resulting in fibrosis that interferes with electrical conduction [25].

IL-12p70, produced primarily by dendritic cells and macrophages, plays a central role in the differentiation of *naïve* T cells toward the Th1 phenotype, promoting the production of IFN-γ and TNF-α by T and natural killer (NK) cells [35]. This IL-12–IFN-γ axis potently activates macrophages and CD8+ T cells, promoting parasite clearance but also contributing to myocardial damage due to persistent inflammation [36]. Sustained elevation of IFN-γ in patients with cardiac forms of the disease induces apoptosis, mitochondrial dysfunction, and oxidative damage, and correlates with higher levels of fibrosis and mononuclear-cell infiltration [6,37]. For their part, IL-2 and IL-15 act as proliferation and survival cytokines for effector T lymphocytes and NK cells. IL-2 stimulates the clonal expansion of CD4+ and CD8+ T cells, while IL-15 contributes to their activation and longevity, especially in chronic inflammatory environments such as Chagas myocardium [38,39]. These interleukins promote the persistence of cytotoxic T lymphocytes in cardiac tissue, which can exacerbate damage in sensitive regions such as the conduction system.

The chemokine MIP1α is also elevated in these patients and plays an essential role in the recruitment of monocytes, macrophages, and lymphocytes to the myocardium [32]. Its expression has been associated with focal areas of inflammation, including in structures of the conduction system, where it can disrupt tissue architecture and impair the propagation of electrical stimulation [40,41].

Finally, IL-10, an anti-inflammatory cytokine produced by regulatory T cells and macrophages, is possibly elevated as a compensatory mechanism against the predominant proinflammatory environment. However, in many cases, this production is insufficient or delayed, allowing tissue damage to progress [42,43]. Taken together, the immunological profile characterized by the statistically significant increase in IL-10, IL-12p70, IL-2, IL-15, MIP1α, and IFN-γ suggests that ventricular conduction disorders in chronic Chagas disease are not only a structural consequence but also an expression of an active and sustained immunopathogenesis that involves multiple pathways of cellular activation, recruitment, and local damage.

Evidence suggests that host immunopathology may be as critical as parasite virulence in determining disease outcomes. We have previously demonstrated an association between circulating anti-M2 and anti-beta1 antibodies and conduction disturbances in CC [8,9].

### Strengths and Limitations

This is one of the first studies to simultaneously evaluate multiple cytokines and their association with EF and IVCD in patients with CC. Our study has several limitations that should be considered when interpreting the findings. First, the sample size was relatively small, particularly for the subgroup analyses. This may have limited the statistical power to detect subtle differences in cytokine levels. Second, the cross-sectional design does not allow causal relationships to be established, nor for determining whether cytokine alterations precede conduction disorders. Longitudinal studies are needed to clarify temporal relationships. Third, although we evaluated 11 cytokines, other relevant immune mediators were not assessed, which may provide additional insights into the mechanisms of early cardiac involvement. Fourth, all participants were recruited from a single tertiary-care center, which may limit the generalizability of the findings. Finally, A causal relationship cannot be established between the preserved LVEF subgroup, and we can only present the results in a purely descriptive manner, taking into account the spectrum restriction due to the exclusion of LVEF 36 to 49 percent. Future studies are needed to explore their causal, prognostic, or therapeutic significance.

Despite these limitations, our findings provide novel insights into the immune alterations associated with conduction disorders during the early stages of CD. They highlight the potential role of early cytokine dysregulation in cardiac involvement and may contribute to the identification of novel biomarkers for disease progression.

## 5. Conclusions

In patients with Chagas disease and preserved EF, the presence of intraventricular conduction disorders appears to be associated with a distinct cytokine profile characterized by elevated IL-10, IL-12p70, IL-2, IL-15, MIP-1α, and IFN-γ. In contrast, no statistically significant associations were observed between cytokine levels and systolic dysfunction. These findings suggest a specific inflammatory profile linked to conduction disturbances, even in the absence of impaired ventricular function. Despite this finding, a causal relationship cannot be established, and future studies are needed to explore their prognostic or therapeutic significance.

## Figures and Tables

**Table 1:** Patient characteristics.

	LVEF < 35% (n = 22)	LVEF > 50% (n = 22)
Age (mean ± SD)	63.4 (±10.8)	55.6 (±11.4)
Male (n, %)	15 (68.2%)	11 (50.0%)
Hypertension (n, %)	18.2	3 (13.6%)
Type 2 diabetes (n, %)	1 (4.5%)	1 (4.5%)
Dyslipidemia (n, %)	1 (4.5%)	3 (13.6%)
Atrial fibrillation (n, %)	3 (13.6%)	1 (4.5%)
Stroke (%)	3 (13.6%)	4 (18.2%)
Blocks, pacemakers and ICD (n, %)	21 (95.5%)	12 (55.5%)
Pacemaker (n, %)	2 (9.1%)	0 (0.0%)
ICD (n, %)	6 (27.3%)	1 (4.5%)
Arrhythmia (n, %)	11 (50.0%)	4 (18.2%)
Use of beta blockers (n, %)	16 (77.3%)	3 (13.6%)
LVEF (mean % ± SD)	28.8 (±4.4)	60.9 (±6.7)

ICD = implantable cardioverter–defibrillator, LVEF = left ventricular ejection fraction, SD = standard deviation.

**Table 2:** Plasma cytokine levels compared by reduced vs. preserved LVEF.

	Cytokine Plasma Level as Median (25th–75th Percentile), in pg/mL		
Cytokine	LVEF < 35% n = 22	LVEF > 50% n = 22	*p*
TNF-α	16.479 (12.921–26.561)	23.886 (17.556–25.942)	0.189
IL 6	1.085 (0.316–3.359)	3.692 (0.256–6.244)	0.145
MCP-1	186.942 (144.672–249.276)	186.646 (142.071–207.473)	0.481
IL-10	19.42 (6.602–25.988)	17.792 (3.292–24.775)	0.607
MIP1α	6.921 (2.091–9.201)	8.236 (0.603–11.135)	0.864
IL-12 p70	9.84 (7.19–13.204)	9.785 (5.422–11.712)	0.642
IL2	2.394 (2.041–2.897)	2.795 (1.763–3.453)	0.481
IL-1β	2.04 (1.324–2.688)	2.034 (1.148–2.62)	0.821
IL-15	4.018 (2.546–7.256)	4.636 (2.684–5.595)	0.724
IL-17A	4.022 (2.961–5.252)	3.681 (1.978–5.024)	0.725
INF-γ	8.726 (6.396–10.74)	7.995 (4.311–9.779)	0.372

LVEF = left ventricular ejection fraction.

**Table 3:** Cytokine levels in patients with preserved LVEF, comparing those with or without conduction disease.

	With CD (Group 1) (n = 10)	Without CD (Group 2) (n = 12)	Hodges–Lehmann Differences (CI 95%)	*p*	n	q
Total patients	10	12				
AGE (mean %, ±SD, YEARS)	61.30 ± 13.36	50.92 ± 7.10	0.107			
Male (n, %)	6 (60)	5 (41.7)	0.392			
LVEF (mean %, ±SD)	59.60 ± 6.28	62.00 ± 7.07	0.539			
	Cytokine plasma levels (pg/mL)					
TNF-α (mean, range)	7.25 (4.09–8.70)	10.83 (9.15–12.92)	−3.94 (−8.42 to −1.74)	0.006	1 #	0.005
IL 6 (mean, range)	9.83 (3.40–18.44)	24.49 (19.45–32.64)	−15.90 (−30.45 to −4.03)	0.008	2 #	0.009
MCP-1 (mean, range)	3.35 (0.94–7.51)	10.33 (6.94–13.42)	−6.27 (−11.07 to −0.30)	0.015	3 #	0.014
IL-10 (mean, range)	3.36 (1.92–4.51)	7.09 (4.07–8.18)	−3.12 (−5.30 to −0.28)	0.02	4 #	0.018
MIP1α (mean, range)	2.07 (1.81–2.61)	2.70 (2.39–3.73)	−0.92 (−1.98 to −0.17)	0.023	5 #	0.023
IL-12 p70 (mean, range)	9.02 (6.43–11.17)	13.20 (9.57–15.53)	−4.41 (−8.10 to −0.36)	0.033	6 #	0.027
IL2 (mean, range)	0.79 (0.21–1.38)	3.31 (1.11–5.45)	−2.19 (−4.81 to 0.00)	0.054	7	0.032
IL-1β (mean, range)	14.53 (12.52–19.49)	22.13 (16.21–28.21)	−4.67 (−13.92 to 1.29)	0.086	8	0.036
IL-15 (mean, range)	3.69 (2.21–4.56)	4.95 (3.40–6.05)	−1.45 (−2.95 to 0.55)	0.106	9	0.041
IL-17A (mean, range)	1.87 (1.43–2.23)	2.62 (1.19–3.63)	−0.75 (−1.76 to 0.64)	0.271	10	0.045
INF-γ (mean, range)	186.94 (172.68–226.13)	194.78 (123.08–330.28)	2.84 (−150.81 to 76.25)	1	11	0.05

CD = Chagas’s disease, LVEF = left ventricular ejection fraction. Cytokine concentrations are expressed as median (25th to 75th percentile). The magnitude of the difference between the groups is expressed as the Hodges–Lehmann position statistic and its corresponding 95% confidence interval. # Only null hypotheses with *p*-value ≤ the q-value calculated using the Benjamini–Hochberg procedure for the false discovery rate were rejected; therefore, differences between groups for cytokines 1 to 6 of the ranking were considered significant.

## Data Availability

The original contributions presented in this study are included in the article/Supplementary Materials. Further inquiries can be directed to the corresponding author.

## Supplementary Materials

The following supporting information can be downloaded at: https://www.mdpi.com/article/10.3390/medsci13040309/s1, Table S1: Complete dataset with the raw data from this study. The identity of the participating patients is coded to protect their privacy.
